# Validity evidence for programmatic assessment in competency-based education

**DOI:** 10.1007/s40037-018-0481-2

**Published:** 2018-11-14

**Authors:** Harold G. J. Bok, Lubberta H. de Jong, Thomas O’Neill, Connor Maxey, Kent G. Hecker

**Affiliations:** 1 Centre for Quality Improvement in Veterinary Education, Faculty of Veterinary Medicine, Utrecht University, Utrecht, The Netherlands; 2 Department of Psychology, University of Calgary, Calgary, Canada; 3 Veterinary Clinical and Diagnostic Sciences, Faculty of Veterinary Medicine, University of Calgary, Calgary, Canada

**Keywords:** Outcome-based education, Competency development, Programmatic assessment, Learning curves, Performance-relevant information

## Abstract

**Introduction:**

Competency-based education (CBE) is now pervasive in health professions education. A foundational principle of CBE is to assess and identify the progression of competency development in students over time. It has been argued that a programmatic approach to assessment in CBE maximizes student learning. The aim of this study is to investigate if programmatic assessment, i. e., a system of assessment, can be used within a CBE framework to track progression of student learning within and across competencies over time.

**Methods:**

Three workplace-based assessment methods were used to measure the same seven competency domains. We performed a retrospective quantitative analysis of 327,974 assessment data points from 16,575 completed assessment forms from 962 students over 124 weeks using both descriptive (visualization) and modelling (inferential) analyses. This included multilevel random coefficient modelling and generalizability theory.

**Results:**

Random coefficient modelling indicated that variance due to differences in inter-student performance was highest (40%). The reliability coefficients of scores from assessment methods ranged from 0.86 to 0.90. Method and competency variance components were in the small-to-moderate range.

**Discussion:**

The current validation evidence provides cause for optimism regarding the explicit development and implementation of a program of assessment within CBE. The majority of the variance in scores appears to be student-related and reliable, supporting the psychometric properties as well as both formative and summative score applications.

## What this paper adds

A pressing issue facing CBE is ensuring the feasible use of robust assessment methodologies that result in valid scores by which to provide feedback on learning and make decisions about learner progression. This study provides validity evidence involving generalization inferences made about the development of competence across domains of interest within and across time and contexts using multiple assessment methods and multiple independent assessors. The inferences and validation evidence reported here provide cause for optimism regarding the explicit development and implementation of a program of assessment within CBE.

## Introduction

A pressing issue facing competency-based education (CBE) is ensuring the feasible use of robust assessment methodologies that result in useful performance-relevant information (PRI) by which to provide feedback on learning and make decisions about learner progression. Longitudinal assessment of competencies in workplace learning environments is new to health professions education [1, 2]. As a result, a model for programmatic assessment has been proposed that simultaneously optimizes assessment for learning and high-stakes decision-making [3, 4]. Programmatic assessment in CBE involves the following characteristics and complexities.

First, it allows the instruction, guidance, supervision, and evaluation of multiple affective, behavioural, and cognitive performance dimensions (e. g., roles, competencies, activities). Second, performance judgements offer the opportunity for student-specific, performance-relevant information. PRI, which is defined as the formal collected information, both quantitative and qualitative (e. g., assessment scores and narrative feedback), that informs student learning within a certain context [5, 6], is important because it contains data involving the assessment of performance over time, and therefore about learning. However, these performance judgements may be affected by more than just PRI (e. g., bias), and therefore understanding the impact of variables such as competency assessed, method of assessment, and the context of performance is vital. Third, a variety of performance indicators (spanning quantitative and qualitative information) should serve as input for performance judgements. Multiple indicators provide PRI from a variety of perspectives, potentially enriching the experience, breadth, and quality of feedback. Fourth, a programmatic approach to assessment allows understanding of within- and between-individual changes over time. Within-individual change of performance provides insight into individuals’ specific growth (or declining) patterns over time. Between-individual change provides insight into the variance of individual learning over time (i. e., the extent to which students follow similar or different learning curves). Recently, the importance of a programmatic approach to assessment has been recognized by the ‘Ottawa Conference Working Group on Good Assessment’ by introducing the term ‘Systems of Assessment’ in their 2018 draft report ‘Consensus Framework for Good Assessment’ [7].

The ideal model for programmatic assessment should both provide ongoing PRI (e. g., narrative feedback from workplace-based assessments) regarding students’ development within predefined competencies and align CBE with curricular program outcomes. In order to use PRI in formative (e. g., providing feedback for performance development) and summative functions (e. g., high-stakes decision-making for promotion, examination, licensure), validation evidence of programmatic assessment scores is required. Inferring validity from observation-based scores is complex, as there are typically multiple competencies, assessment methods, raters, contexts, and time points involved [8, 9]. Kane’s validity framework offers a structure to gather information regarding the quality of assessment procedures by treating validity as involving a series of four inferences drawn from observed scores and considers whether those inferences are justified [10, 11]. These four inferences, for which sufficient validity arguments must be provided, include scoring, generalization, extrapolation, and implications [12]. Each validity argument is underpinned by a coherent analysis of evidence in support of (or against) the interpretations of test score meaning [11, 13]. In this paper, we focus on the generalization inference made about students’ observed clinical performance scores over time. This is important because solid arguments involving this inference are needed before investigating extrapolation and implications inferences. We further the work published in health professions education about workplace-based assessment and provide generalization evidence which ‘includes observations that can vary in a number of ways, involving, for example, samples of tasks, testing contexts, occasions in which the test is administered, and possibly raters who score the responses’ [11].

Despite existing and future transformations in healthcare curricula toward CBE and programmatic approaches to assessment, still no large-scale longitudinal multi-method and multi-competency investigations exist. This is important because the claims advanced by CBE and programmatic assessment are predicated on the assumption of valid measurement. Ultimately, evidence in support of (or against) the inferences made about students’ clinical performance levels over time is required (i. e., the validity of competency-based scores). We performed a retrospective quantitative analysis of 327,974 individual assessment data points from 16,575 completed assessment forms involving 962 students over multiple time periods using both descriptive (visualization) and modelling (inferential) analyses. To investigate if programmatic assessment can be used within a CBE framework to track progression of students learning within and across competencies over time we tested the following hypotheses:

### Hypothesis 1

Performance scores collected over time should be reliable.

### Hypothesis 2

Overall aggregate performance scores should increase over time to demonstrate learning (i. e., the progression of learning should be clear through visualization as well as positive slopes in empirical modelling).

### Hypothesis 3

The dominant source of systematic variance in performance ratings should be attributable to inter-student differences in performance levels.

### Hypothesis 4

Scores from multiple competencies from multiple assessment methods should differentiate performance over time.

## Methods

### Setting

The Faculty of Veterinary Medicine, Utrecht University (FVMU) in the Netherlands was established in 1821 and offers a 6-year program that consists of 3 years of preclinical education and 3 years of clinical education. In 2010 FVMU renewed the final 3 years of the program and designed and implemented a 3-year competency-based clinical curriculum with a programmatic approach to assessment resulting in the degree of doctor in veterinary medicine. Each year 225 students enrol into their first year. The 3‑year clinical program is organized around clinical rotations in disciplines related to three separate tracks: equine health, companion animal health, and farm animal health. Apart from general rotations in different clinical departments, students mainly undertake rotations in disciplines related to their chosen track. While working side by side with clinical staff, students encounter a variety of learning activities during their clinical rotations. At FVMU teaching faculty are offered a 2-year development program to attain a basic teaching qualification, including specific courses related to the program outcomes and the applied assessment procedures.

### Competency-based education and programmatic assessment

**Fig. 1:**
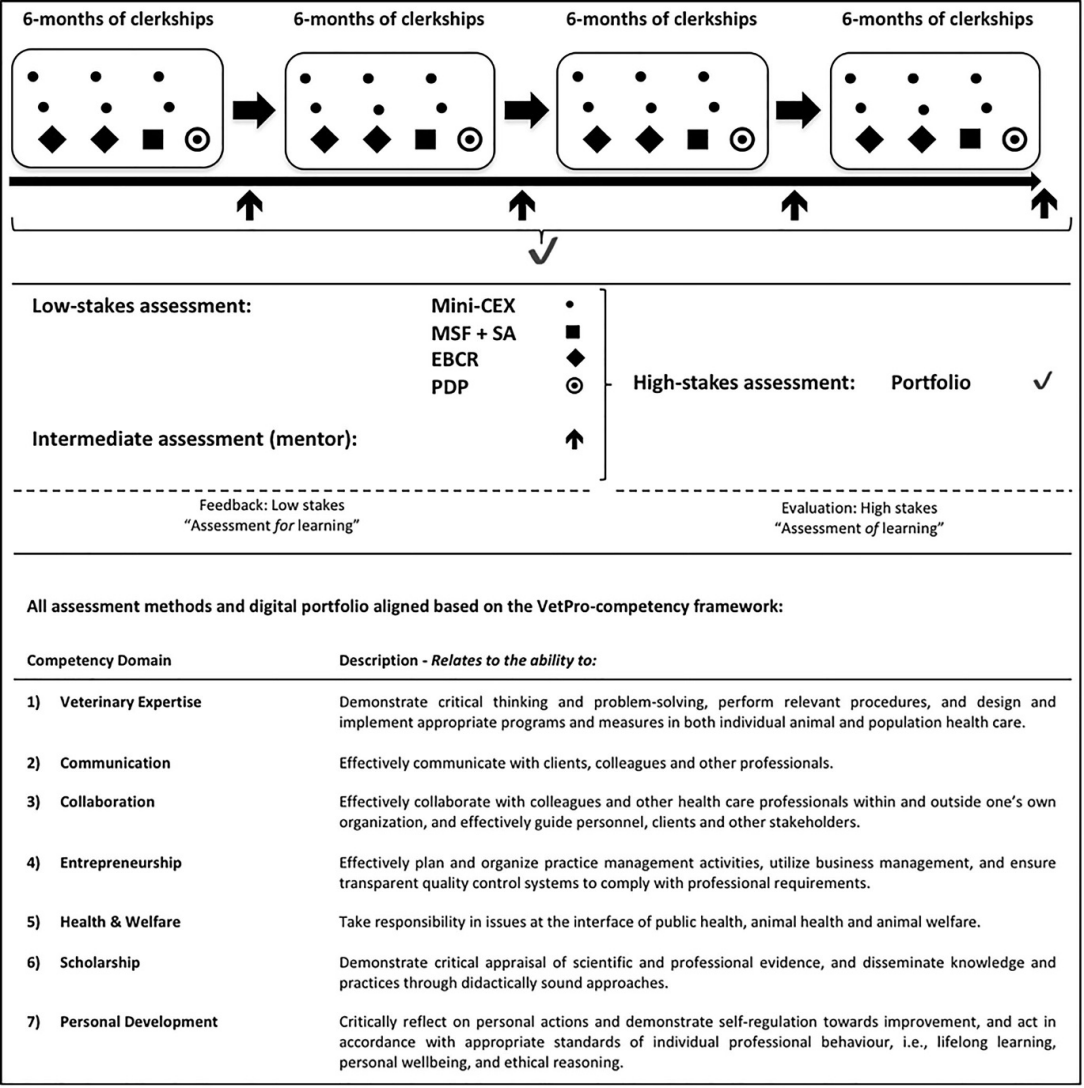
Schematic overview of competency-based assessment program at the Faculty of Veterinary Medicine, Utrecht University. *Mini-CEX* mini clinical evaluation exercise, *MSF* multisource feedback, *SA* self-assessment, *EBCR* evidence-based case report, *PDP* personal development plan

During clinical rotations, each student is required to collect workplace-based assessments (WBA) based on performance observations. The WBAs are organized around competency domains that are described in the VetPro-competency framework (see Fig. 1; [14–16]). The assessment information is collected in a digital portfolio for which the student bears responsibility. Supervisors are not informed about students’ previous performance on WBAs while observing and assessing clinical performance. Twice a year each student has an individual student-mentor meeting to discuss progress and formulate new learning goals for the upcoming period. After 2 years (maximum program duration: 124 weeks) and at the end of the program a high-stake decision is made by two independent members of an assessment committee for promotion or licensure purposes based on the judgement of a multitude of individual WBAs in a digital portfolio [17]. The digital portfolio aggregates and visualizes assessment data for each competency domain and assessment method across rotations over time. As in the first 2 years of clinical training the educational program is comparable in design for all three tracks, in this study WBA data collected during rotations in this period were used (see Fig. 1 for a schematic overview of the assessment program at FVMU). The scores, on a 5-point Likert-scale (1 = novice; 5 = expert) derived from the mini clinical evaluation exercise (Mini-CEX), the multisource feedback instrument (MSF), and the self-assessment were included. The rationale for limiting data analysis to these WBAs is that they are most frequently used in all rotations, provide data on all seven competency domains and are applied in all three tracks. The mini-CEX contains 11 items and is applied by supervisors to provide PRI based on direct observation of students’ performance. The MSF instrument is used by a variety of assessors (e. g., clinicians, veterinary nurses, clients, patient owners) to provide PRI after a prolonged period (>1 week) of observed performance. It contains multiple items for each competency domain with a total maximum of 40 items. The self-assessment contains 28 items and is completed in conjunction with each MSF round.

### Sample

A retrospective quantitative analysis of 327,974 individual assessment data points from 16,575 assessment methods collected by 962 students was performed. The students consisted of *n* *=* 546 companion animal health track students, *n* *=* 291 farm animal health track students and *n* *=* 125 equine health track students. Tab. 1 provides insight into the number of data points analyzed in this longitudinal, multi-method study.

**Table 1:** Assessment methods, domain and item scores included from 1 January 2012 until 6 July 2016

		Total	Included (≤T124)
Assessment methods	Total	17,991	16,575
	Mini-CEX	8,013	7,899
	MSF	8,787	7,514
	SA	1,191	1,162
Domain scores	Total	134,938	124,649
	Mini-CEX	59,362	58,595
	MSF	67,160	57,838
	SA	8,416	8,216
Item scores	Total	363,526	327,974
	Mini-CEX	89,416	88,267
	MSF	241,604	207,952
	SA	32,506	31,755

The total number of assessment data points collected in the program of assessment compared with those analyzed in the study. Difference are due to some students exceeding the maximum length of program (e. g., due to remediation, sickness)

*Mini-CEX* mini clinical evaluation exercise, *MSF* multisource feedback, *SA* self-assessment

### Analyses

Assessment data from identifiable Mini-CEX, MSF, and self-assessment forms between 1 January 2012 and 6 July 2016 were used in the analyses. For each student, the first week with an assessment score represented Time 1 (T1). Scores were then chronologically sorted based by week of assessment (Tx, max. T124). The statistical programs R (R version 3.4.1 (2017-06-30)) and SPSS v24 (IBM, Chicago, IL, USA) were used to analyze the transformed dataset. Generalizability theory was used to calculate reliability coefficients for each assessment method using a nested design where student was crossed with competency nested within week (hypothesis 1). For hypothesis 2 visualizations of the learning curves dependent of student and descriptive statistics were performed using R (package ‘ggplot2’ R). A 4‑level model was run in SPSS using the ‘MIXED’ function with respect to hypotheses 2, 3, and 4.

### Multilevel random coefficient model

The data structure were in a disaggregated, ‘long format’ [18] comprising individual repeated observed scores/ratings (Level 1) nested within competency domains (Level 2), nested within assessment methods (Level 3), nested within students (Level 4). Multilevel models are typically employed with these types of nested educational data as illustrated by Peugh et al. [19]. A multilevel random coefficient model, estimated with restricted maximum likelihood, provides a variance component at each of these four levels, the residual (repeated measures), competency, method, and student components, respectively. Moreover, at the student level (i. e., Level 4), ‘Week of assessment’ was included (with 124 levels pertaining to the student’s week in program) to capture the average student learning trajectory as well as variance in student-specific learning intercepts and slopes. This allows us to model three variance components at this level: student-specific intercept variance (do students start at the same or different performance scores?), student-specific slope variance (do students demonstrate learning at the same or different rates over time?), and their covariance. Overall this multilevel random coefficient model provides an indication of whether scores differentiate students, whether scores change over time, whether we can differentiate competencies and whether assessment methods contribute to variance of observed scores.

Given that the scores collected over time may be non-linear (e. g., sigmoidal-shaped learning curve [20, 21]), models were fit with linear, quadratic, and cubic time functions. Changes in model fit were assessed by the likelihood ratio test using the −2 log likelihood values [22]. Variability in performance could also be due to student self-declared ‘track’ which includes companion animal health, farm animal health, and equine health. This variable was tested as independent categorical covariate variable to determine if it improved model fit.

### Ethical considerations

The Ethical Review Board of the Netherlands Association for Medical Education approved this study (NERB number 884).

## Results

The reliability for the Mini-CEX, MSF, and self-assessment scores were *Ep*^*2*^ = 0.86, 0.88, and 0.90 respectively, indicating that scores for students were consistent across weeks and competencies (Tab. 2). For all three methods, considerably more variance was accounted for by student nested within week (ranging from 41.98 to 45.35%), compared with week (ranging from 3.95 to 11.49%) and competency nested within week (ranging from 2.89 to 6.89%).

**Table 2:** Generalizability analysis by method

Source	MSF		Mini-CEX		SA	
	σ^2^	%	σ^2^	%	σ^2^	%
Week (w)	0.02	3.95	0.06	11.49	0.03	10.85
Student(s)|week	0.16	43.16	0.21	41.98	0.12	45.35
Competency (c)|week	0.01	2.89	0.03	5.35	0.02	6.98
s|w*c|w, error	0.19	50.00	0.21	41.19	0.10	36.82
Total	0.38		0.51		0.26	
G-coefficient (Ep^2^)	0.86		0.88		0.90	

σ^2-^ variance component, % percent, G‑coefficient = $\sigma _{\left(s\mid w\right)}^{2}/\left((\sigma _{\left(s\mid w\right)}^{2}+\left((\frac{\sigma _{\left(s\mid w*c\mid w,e\right)}^{2}}{n_{c}}\right)\right)$

Multisource feedback (*MSF*), mini clinical evaluation exercise (*Mini-CEX*), self-assessment (*SA*) for week (*n* = 124), student nested within week (*n* = 962 students), and competency nested within week (*n* = 7 competency domains).

**Fig. 2:**
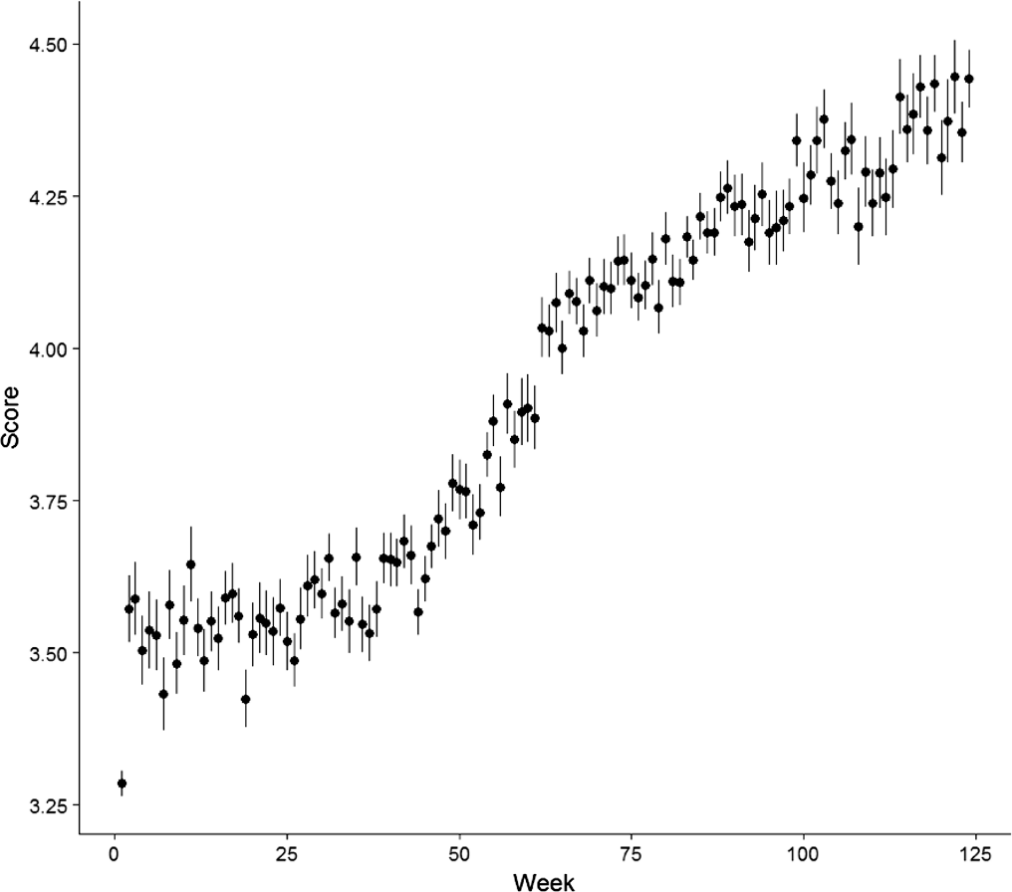
Development of performance (score) dependent of student. The Y‑axis represents the average score of students’ performance per week on a 5-point Likert-scale. The average score per week is collapsed per competency domain, per method and per student. The X‑axis represents 124 weeks of clinical training. The error bars represent the standard error (SE)

The learning curve in Fig. 2 represents the mean scores over time (week of assessment) collapsed across student, method, and competency domain (*µ* = 3.29 in week 1 to *µ* = 4.44 in week 124). The learning curve suggests a cubic, i. e., sigmoidal, shape to the data.

**Fig. 3:**
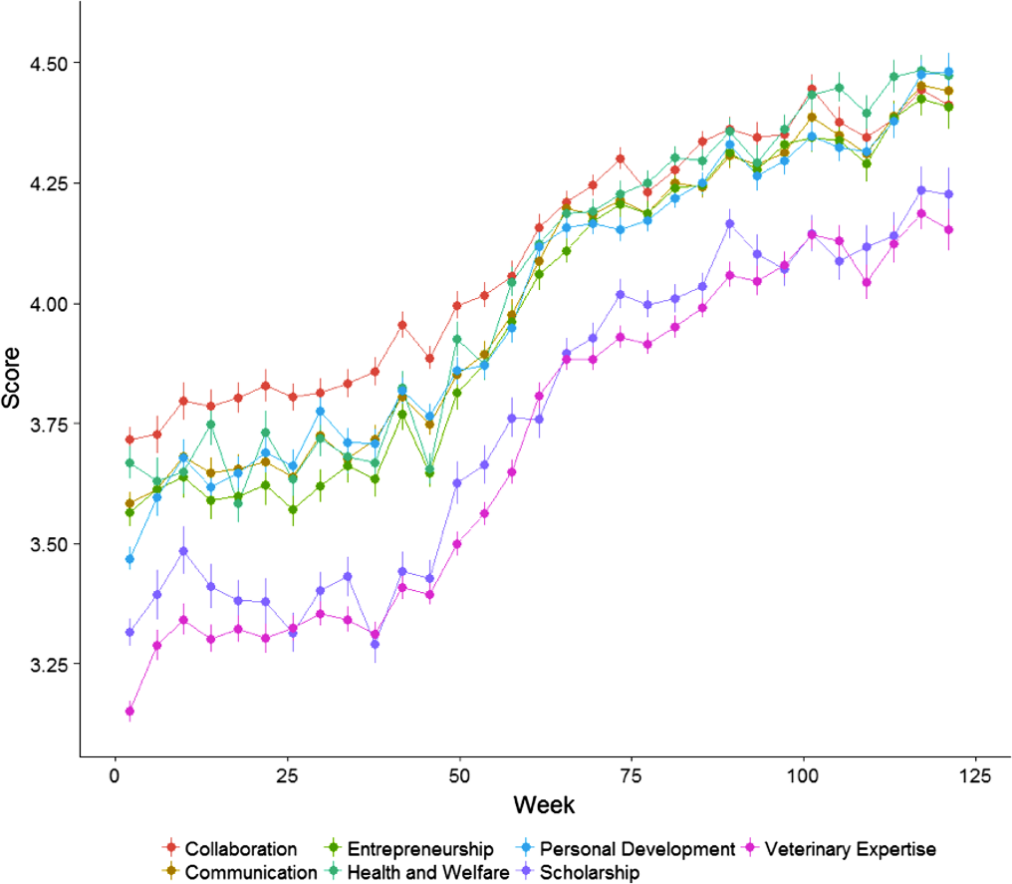
Average competency domain score (µ, se) within student across competency domain discretized by four weeks. Y‑axis represents the average score of students’ performance per competency domain on a 5-point Likert-scale. The average score is collapsed per method and per student. The error bars represent the standard error (SE)

Fig. 3 illustrates progression of performance within competency domain. Progression appears to be sigmoidal across all domain scores with scores from veterinary expertise and scholarship being consistently lower than scores from other competency domains.

Tab. 3 contains the results of the variance components estimated in the multilevel random coefficient model. To assess the effect of time we assessed model fit with trajectories of performance being linear or nonlinear (i. e., quadratic or cubic) over time [18]. Model 1 is the baseline model that does not include any fixed effect involving time, but it includes all variance components. Model 2 includes a linear fixed effect involving time, Model 3 includes both linear and quadratic (one inflection point) effects involving time and Model 4 includes linear, quadratic and cubic (s-shaped—two inflection points) effects involving time. Apart from time, each model contains the same random coefficient structure. Level 1 contains the residual variance (repeated measures and non-systematic variance), Level 2 contains the variance due to competency domain, Level 3 contains the variance due to assessment method, and Level 4 contains the variance due to student-specific intercepts (i. e., starting points), student-specific slopes (change over time/trajectories), and the covariance of student-specific intercepts and slopes.

**Table 3:** Multilevel random coefficient models, where repeated measures (residual, Level 1) are nested within Competency Domain (Level 2), Assessment Methods (Level 3) and Students (Level 4). Model 2–4 assess the effect of linear and non-linear time (inflection points, cubic and quadratic) on repeated measures

Parameter Effects	Model 1 (SE)	% of variance	Model 2 (SE)	% of variance	Model 3 (SE)	% of variance	Model 4 (SE)	% of variance
*Fixed effects*								
Intercept	3.876 (0.009)*		3.487 (0.014)*		3.405 (0.015)*		3.487 (0.015)*	
Week			0.008 (0.0002)*		0.0122 (0.003)*		0.002 (0.001)*	
Week * Week					−3.88 × 10^−5^ (1.9 × 10^−6^)*		0.0002 (1.9 × 10^−5^)*	
Week * Week * Week							−1.17 × 10^−6^ (5.4 × 10^−8^)*	
*Random effects*								
Level 1: Repeated Measures (Residual)	0.308 (0.001)*	46.53	0.308 (0.001)*	61.11	0.307 (0.001)*	59.73	0.306 (0.001)*	60.41
Level 2: Competency Domain (Intercept)	0.014 (0.001)*	2.11	0.014 (0.001)*	2.78	0.014 (0.001)*	2.72	0.014 (0.001)*	2.72
Level 3: Assessment Method (Intercept)	0.073 (0.003)*	11.03	0.068 (0.003)*	13.49	0.069 (0.003)*	13.42	0.065 (0.003)*	12.90
Level 4: Student (Intercept)	0.267 (0.020)*	40.33	0.114 (0.008)*	22.62	0.124 (0.009)*	24.12	0.122 (0.008)*	23.98
Level 4: Student (Covariance)	−0.005 (0.0003)*		−0.002 (0.0001)*		−0.002 (0.0001)*		−0.002 (0.001)*	
Level 4: Student (Slope)	9.53 × 10^−5^ (5.0 × 10^−6^)*	0	2.61 × 10^−5^ (1.7 × 10^−6^)*	0	2.76 × 10^−5^ (1.8 × 10^−6^)*	0	2.57 × 10^−5^ (1.7 × 10^−6^)*	0
−2 Loglikelihood	219,200.57		218,308.86		217,907.54		217,488.23	
Degrees of Freedom (DF)	7		8		9		10	

*SE* standard error*, %* percent

*P *<* *0.001*

In Model 1, without the fixed effect of time being modelled, most of the variance at Level 4 (student) was due to different intercepts (40.33%), suggesting that students have different starting points corresponding to general levels of achievement. However, the rate of learning does not differ across students as the slope coefficient accounted for 0.00% of the variance in performance scores. The covariance term was significant and in the negative direction, suggesting a higher starting point is associated with a less positive rate of change over time, but the effect size was negligible (−0.0005). The variance associated with Level 3 (assessment method) was 11.03%, whereas the variance associated with Level 2 (competency) was 2.11%. In other words, more variance in performance is due to the method of assessment compared with the assessment dimension, suggesting that the same competency measured using different methods affects ratings more than different competencies measured with the same method. The final variance component is at Level 1, which is the variance across repeated measures nested within domain/method/student including the residual (46.53%).

Model 2 includes time as a linear function which models change in individual performance as constant (straight line) over time. Including the effect of linear time (week) as a fixed effect significantly improves model fit (χ^2^ (1) = 219,200.57 − 218,308.86 = 891.71, *p* < 0.01). The fixed effect of time as a linear (slope) component was 0.008 (*p* <0.001). This is the average student trajectory over time or the expected change over one of the 124 units of time for a given student. It illustrates that for each week that passes the predicted average student increase in performance is 0.008. The mean estimated performance score was 3.49. Given that there are 124 weeks coded, from the beginning to the end of the training program, the expected average student increase in performance is 0.992. As performance scores were largely between 3.2 and 4.5 (Fig. 2), this corresponds to a large effect of learning over time in program.

Between Model 1 and 2 there was a decrease of variance between students (Level 4) of 0.153 (0.267–0.114) suggesting that 15.3% of the between student variance is associated with change over linear time. Apart from this change the general trends remain the same, most of the variance at Level 4 (student) was due to different intercepts (22.62%), suggesting that students have different starting points and levels of achievement even when linear time is accounted for. The rate of learning does not differ across students as the slope coefficient accounted for 0.00% of the variance in performance scores. The covariance term again was also significant and in the negative direction, suggesting a higher starting point is associated with a less positive rate of change over time, but the effect size was negligible (−0.0002). The variance associated with Level 3 (assessment method) increases to 13.49%, whereas the variance associated with Level 2 (competency) was 2.78%. The final variance component is at Level 1, which is the variance across repeated measures nested within domain/method/student including the residual (61.11%).

Model 3 and Model 4 assess if nonlinear functions of time (curvatures in performance trajectories) improve model fit. The quadratic model (Model 3) improved model fit over the linear model (χ^2^ (1) = 218,308.86 − 217,907.54 = 401.32, *p* < 0.01). There was a significant linear effect (β = 0.0122, SE = 0.003, *p* < 0.001) and a significant quadratic effect (β = −3.88 × 10^−5^, SE = 1.9 × 10^−6^, *p* < 0.001), therefore both are retained suggesting that nonlinear trajectories fit the data. The cubic model (Model 4) again improves model fit (χ^*2*^ (1) = 217,907.54 − 217,488.23 = 419.31, *p* < 0.01). There was a significant linear effect (β = 0.002, SE = 0.001, *p* < 0.001), a significant quadratic effect (β = 0.0002, SE = 1.9 × 10^−5^, *p* < 0.001), and a significant cubic effect (β = −1.17 × 10^−5^, SE = 5.4 × 10^−8^, *p* < 0.001). All are retained in the model. This provides evidence of a sigmoidal curve where learning accelerates around week 40 (quadratic effect, positive β value) then starts to level off around week 75 (cubic effect, negative β value).

The trends for Model 4 are the same as Model 2. Where most of the variance at Level 4 (student) was due to different intercepts (23.98%). The rate of learning does not differ across students as the slope coefficient accounted for 0.00% of the variance in performance scores. The covariance term was again significant and negative (−0.0002). The variance associated with Level 3 (assessment method) was 12.90%, whereas the variance associated with Level 2 (competency) was 2.72%. Indicating again, more variance in performance is due to the method of assessment compared with the assessment dimension.

Once Model 4 was built we assessed the effect of track. There were non-significant fixed effects involving track (χ^2^ (2) = 217,488.23 − 217,479.24 = 8.99, *p* = n. s.) meaning no differences across student interest. Including this variable does not increase model fit providing evidence that we can combine data across tracks, i. e., derived from different learning environments.

## Discussion

The major findings from this study are: 1) Scores from each assessment method over time demonstrate reliability, 2) Linear, quadratic, and cubic functions support the positive development of competencies over time, 3) The majority of the variance in scores is student-related, and 4) Variance in performance is more due to the method of assessment than to the competency assessed (but most of all to inter-individual student proficiency levels). The results provide validity arguments for Kane’s inferences made regarding generalization.

The inferences and validation evidence reported on here provide cause for optimism regarding the explicit development and implementation of a program of assessment, i. e., system of assessment, within CBE [4, 7]. The majority of the variance in scores appears to be student-related and reliable, thereby supporting the psychometric properties as well as both formative and summative score applications. The learning curve provides an argument for applying assessment scores to track clinical performance and learning as a result of the assessment program that was created to collect, reflect, and provide PRI to both the student and the faculty. This provides support for a mastery approach to learning in outcome-based health professions education as suggested in previous literature about training and assessment of future healthcare professionals [2, 23, 24]. The fact that students’ interest, i. e., chosen track, did not increase model fit supports our claim related to the generalization inferences. It provides validity evidence to the argument for the generalizability and usability of PRI and the implemented program of assessment in outcome-based health professions education. The analyses of PRI in programs of assessment could help to provide transparent decision processes, determine when enough information is available to reliably assess clinical competence, and may be useful in exploring new psychometric models [9].

Given that the majority of variance was student-related, it is apparent that CBE can identify and quantify inter-student differences in performance. As this is the primary purpose of CBE (i. e., to accurately differentiate performance levels), it is encouraging for the validity of CBE programs and their future adoption. Importantly, the rate of learning did not differ across students as indicated by negligible student level slope variance. This suggests that *all students benefitted equally* from engagement in the CBE program, rather than some benefitting more than others. Method- and competency-related sources of variance were the next most impactful. Indeed, this is also common in workplace literature in which competency-based measurement has existed for decades [25]. The method of measurement, in our view, provides unique and valuable incremental perspectives on student competency levels. A very large method component would be undesirable, however, as all variance in competency measurement should not be method-based. However, we interpret the non-trivial method component found in this research is supportive of using multiple assessment methods. Competencies are not routinely found to account for a large source of variance in achievement levels [26], but they are important for capturing a student’s profile across performance areas. Indeed, for formative feedback, they provide the student with relative strengths and development areas, so that actions for improvement can be focused. Moreover, competency variance was large enough to be meaningfully detected, which supports their continued use.

### Limitations

In this study, we examined validity evidence related to the generalization inferences as described in Kane’s validity framework. However, a first limitation of this study is that we did not investigate Kane’s scoring, extrapolation, or implementation inferences. Future research should focus on providing evidence to examine the validity argument in support of (or against) these inferences illustrating how observations relate to scores (i. e., scoring inference), and how assessment scores relate to performance in the real world (i. e., extrapolation inference) and how the high-stakes performance decision impacts the individual, the curriculum, and society (i. e., implication inference) [12].

Secondly, while the data support the generalizations made from the scores of the assessment program implemented at FVMU (see Fig. 1), much is still unknown about the key design and implementation issues of successful programs of assessment in CBE. For instance, questions that need to be answered relate to features of the learning environment that contribute to the reliability of performance scores; relate to how to effectively train raters, i. e., to minimize rater biases; relate to exploring methods of measuring competency development, i. e., effectively provide PRI, that gives the most utility; relate to how to optimize the formative function of PRI in programs of assessment in CBE; and relate to how to effectively apply PRI in making summative decision-making judgements. With respect to this last point, provisional evidence is provided that supports the claim for making accurate high-stakes decisions based on the aggregation of PRI, containing both quantitative data and the necessary high-quality descriptive information, collected by the program of assessment at FVMU. [17]. Further studies and other methodologies are required to assess how best to combine both numerical and qualitative information in programs of assessment for further validity evidence and, more important, to identify the key pieces of information from these rich datasets for the assessment for learning and for high-stakes decisions.

Finally, further insight is required regarding if an ideal, generalizable set of competencies should be reflected in a CBE context. Competencies are often dominated by a ‘halo’ effect in the workplace literature, in which raters use a global impression to drive performance ratings rather than allowing for competency variance [27]. To the extent that a global rating dominates, the appropriateness and utility of assessing competencies may be called into question. We found small competency variance components, and therefore future research should investigate whether particular generalizable taxonomies can be created that lead to higher competency variance in CBE performance scores. Furthermore, it will be of interest to compare our results to other analyses from competency-based frameworks, e. g., entrustable professional activities [28], that utilize a programmatic approach to assessment.

## Conclusion

This study provides validity evidence involving inferences made about students’ clinical performance scores in programmatic assessment. We applied a program of assessment in a competency-based clinical curriculum focused on integrating learning and assessment, and the results support our claims that it helps students to maximize their learning (formative function) and simultaneously allow robust summative decision-making (summative function). Accordingly, learning analytics help to provide an overarching synthesis of PRI to monitor competence development over time, both for formative, summative, and quality control purposes.
